# Record of Meteorological Conditions at Buffalo, for Sept., 1855

**Published:** 1855-11

**Authors:** Sanford B. Hunt


					﻿ART. XV. — Record of Meteorological Conditions at Buffalo, for Sept.,
1855. By Sanford B. Hunt, M. D.
cd
«
<1
S
a
a
•amjajaduiaj,
fo aSuna jsapsaJn	Cl - « x oom o<o h o	cio Clm io o
1—1 ___ r-l__r-l —-I r-l d__r-1	r-l ' r-l d UP
•An	^oct m o>o M m	h m	mommxtci.';-#
-nnrnin	tthstu	m H CO CO M CO O IO tO 00	H 00	t-CT5CPOOCOrrCP-H
-pnntiH	unapt_____________oo co go oo i- t- oo oo oo go	t- r-	1-1-00001-001-05
S	■?UI°<I	. m x co oi m q. -f t~«com’Cjcoi'toHH
5	.moq unaj\'	-p< c> -p« os' d cs op co t-’ o oo' cd	o	i- d	r-i cd	rf	o	oo
q	,__________________cgcocgcoup-piupcacDupcp-ri_____________up	up co	co -p<	-p<	up	up_
•ajtljn	CP . i— i- 1— I— _ CO i— cP t—	i—	CO CO	CO ip	I—
-Jadtuaj, unapt	ajoo-Hupdi—t—r-id-^igdr-i	od	up’ oo	up’ cd	—<	h	d
____________________________co co t — i— co us co r— i — up —r up_up	co co	co up	up	up	co
oocsrrdcoooo ci- i -	ci o:	f o ci o-t'i-d’rr
.	■Ximnnnii	upcocococpupco —< ■—i<c	coi-	cpc-o i-cp - i- - —
u	A4‘L’‘UUIH	00 00 00 00 00 00 000505 00	00 00	00 00	00 GO 00 t— 00 00 CP
S	-iuio-t Aian	*"■• .	.cpoj-d*	,o>	« h	ci cc cp cc m > ci
°	• a u	t—ot-iodcdocdcdcdoioo ddcd'tdcpup’cdt-
•	&_______________________UP CP CO I— UPUPCPCPCPrj<Cpr^ UP UP CD CO UP rfi rf up 1 iP
s £•	■
•	•ajtiQ.duiaT.	.............................. .............
Ph	‘	mvsw CC C) L'i O O CO T!<	CO CD GO O GO O rH o
______________________go go r- iq lq <0 t-~	to ir, o o u; u; ^o up
.05 • < — •
o	» «2 p £ ^^cQtzi^^^ajai
H	r-i O d UP o cd r-i pi UP CP —i r-i	r-i r-l" r-i CP T# d —i r-i r-i
<3	-------------------------■-—-----------------------------------------—-
h	spnor) 10 lui y	00000 o o o o o go otooooooouo
& ______________1__________________	»—«__r-H	r—< r—f_________________t—I	r-l_TH_____________________
#	O^OOO^GOOOOlOI^L^ I—(T^GQCQT^rHr-iOOCQOC^COOOiOtQC^CN GO
H	-AiiniiirntT 5?S2^S?J2S?S?2S^upuPi—cprsicQCOt-OOi—d^CDr-i ^i dp r-i <— co d O up
Pr	. A4lPSluuH CO 0000001— t— JOOOt—CDC— I- UPCOC—t— C—OOUPCOCOI— CDt—C—CPCDi- I- C5 t—
d.	S _____!_____
f=	t
£ "JtltOJ Alaa	. °5.	lrt. 00 00 rf< <fl t- 03 rf rt< 00 UP CP i- CO t- . CP C5 CP CP UP CO CP
O	■	J2r^^PCOUPUP--r-(f-0^005r-5dcdOt^i?^UP—HC0i<05UPC0d05UPO	o
. rf--------t*^mcococpi»N<ogcpt»ii'mcocpt~’»nTi'inin-^m<ao-» -r up cp	co
W?
ffl -ajurduia.r • • • ................................................
•	cot'd'^iocpt'NmmTi'fflcococi’fftciinrtCOionccPomoco-^ cs
—	H f H H H O O H	>G O GG G2 GO O H iG tG O GD GO
Mi-i	..£» ..	£ .m . T S
r°§o°i!	02 02 02	02 02 02	^^02 02 02 ^^02'02*
Q F	.............................. ...
________	^J-h’ CO	H QW M	rl‘ h pj ^4 co CC	ci
spn°[3jo?4uiy_______________________________________________________c-oir:oooooooot- o^ cwov.o o o
~~o oj o h ^o q go oo h	^ooor^cji>u:c0
.	•XiiDiuinn	r-too-c^ooooooTfm	co	t” iq o CQ	co
£	OOOOGOC^QOGOOOOi^QO	00	1^ GO CO GO	GO GO GO
S	-antoj	«MT	c?tc. CP^^**	. CQ CO cp t- cp	d	d t- i- cp	d —
•	o	^u.	q o go v. 7: r- i-T o go uo	riot^^ioairio
£n	GOGOGDGOtQxFlOGOGOGOCQCQ	kOUOLOGO^TTO'^LO
.	t>>	— '	----------—
<; ffi .	,
„ ain^auidj,	o^aSco^co^cooi^GDr-? r^cocoodoi'^Goo
i> _________________________ 1—	GO GO 1^	GO	GO	GO GO	GO	_________tQ 1Q	GO GO	tQ	GO
3 s^g. *	h
& g g	O £	F	02 02	]Zi 02’	02 02	ki 02	02 02	02
” Q ?	.......................... ......
___	r-l	Ci d —	r- rT	-H	d	r-l	 r-l r—I	rl CP	CP	O O	CP   ‘	,
spno[3 jo	l.cuy_o	o o o	o	r-< ic-	cs o	o	o	o_o up	o o	rt<	o o	o
•	•niorncT m Trr	CPCPCPCPCPCPCPCPddupuP	cpup-f-DH-?^
•H luuana JO uj	o’ o- o’ o‘ q o‘ o O O O O o’ O O O <75 o o o o
£ __________________________CPCPCPCPCPCPCPCPCPCPCPCP	CPCPCPCPdCPCPCPCP
<!	CP	r-l	UP	O	-q<	CT5
O	t-	CO	r-l	O	CO	r-l
•tn^HJO 'UJ	cp	aj	ri	d	cd	d
________________________________ '_____________2___________________1_ri_w
•mtirr r-< d CP UP CC t- 00 CP O r-l d CO	UP C® IP- 00 C5O r-l d CP Tf UP CO t- 00 C5 O |
_____________ 4 Ct_____________—I -I rl —I —I rH —I rl — rl d d d d d d d d d d CO I
				

